# Bullous pemphigoid developed after dramatic improvement of severe prurigo nodularis^☆^

**DOI:** 10.1016/j.abd.2022.11.003

**Published:** 2023-05-22

**Authors:** Tomoko Hiraiwa, Natsuko Matsumura, Tatsuhiko Mori, Nobuyuki Kikuchi, Toshiyuki Yamamoto

**Affiliations:** Department of Dermatology, Fukushima Medical University, Fukushima, Japan

Dear Editor,

A 40-year-old female with oral allergy syndrome visited us, complaining of itchy eruptions which appeared one year previously. Physical examination showed a number of firm nodules on the trunk and extremities (Fig. 1). Moreover, subungual hyperkeratosis was observed. Histological examination revealed irregular epidermal proliferation, mild infiltration of mononuclear cells, and fibrosis of the upper dermis (Fig. 2). Laboratory examination showed a white blood cell count of 9700 μL (12% eosinophils), and elevated AST (63 IU/L) and ALT (62 IU/L). The serum level of IgE was over 5,000 IU/mL, whereas anti-BP180 NC16A Ig was within normal range. The patient was treated with various therapies, all of which were disappointing. However, the nodular lesions dramatically improved when she visited our hospital two years later (Fig. 3). She stated that she experienced divorce, which was suspected to release mental stress and led to favorable effects on her skin conditions. Serum IgE level remained elevated (3614 IU/mL). Three and half years after the complete resolution, she re-visited our department, complaining of itchy erythema and a number of bullous lesions. Physical examination showed tense blisters, erosions, and edematous erythema on the upper extremities and back (Fig. 4). Laboratory tests showed an increased number of eosinophils (10%) in the peripheral blood, and serum titer of the anti-BP180 NC16A Ig antibody was over 10,000 U/mL (cut-off < 10). A biopsy from one of the blisters revealed subepidermal blister formation with prominent eosinophil infiltration (Fig. 2). Direct immunofluorescence showed linear deposition of IgG and C3 at the basement membrane zone (Fig. 2), and 1M NaCl-split skin indirect immunofluorescence revealed circulating IgG antibodies reacting on the roof of the cleavage. She was treated with methylprednisolone pulse therapy (1,000 mg for three consecutive days) followed by oral prednisolone, methotrexate, cyclosporine, and plasma exchange therapy. Immunohistological examination revealed BB1-positive basophils in both Prurigo Nodularis (PN) and Bullous Pemphigoid (BP) lesions (Fig. 2). Immunostained cells were counted in random 10 fields under high magnification (×400) of a light microscope, which showed 13.8 ± 5.2 in prurigo nodularis versus 15.2 ± 5.0 in BP lesions.

**Figure 1:**
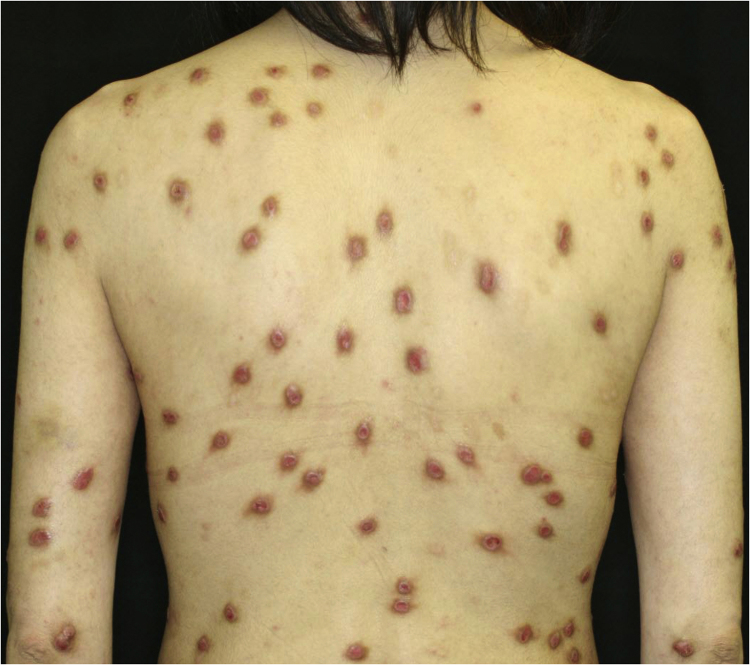
A number of nodular prurigo on the back

**Figure 2:**
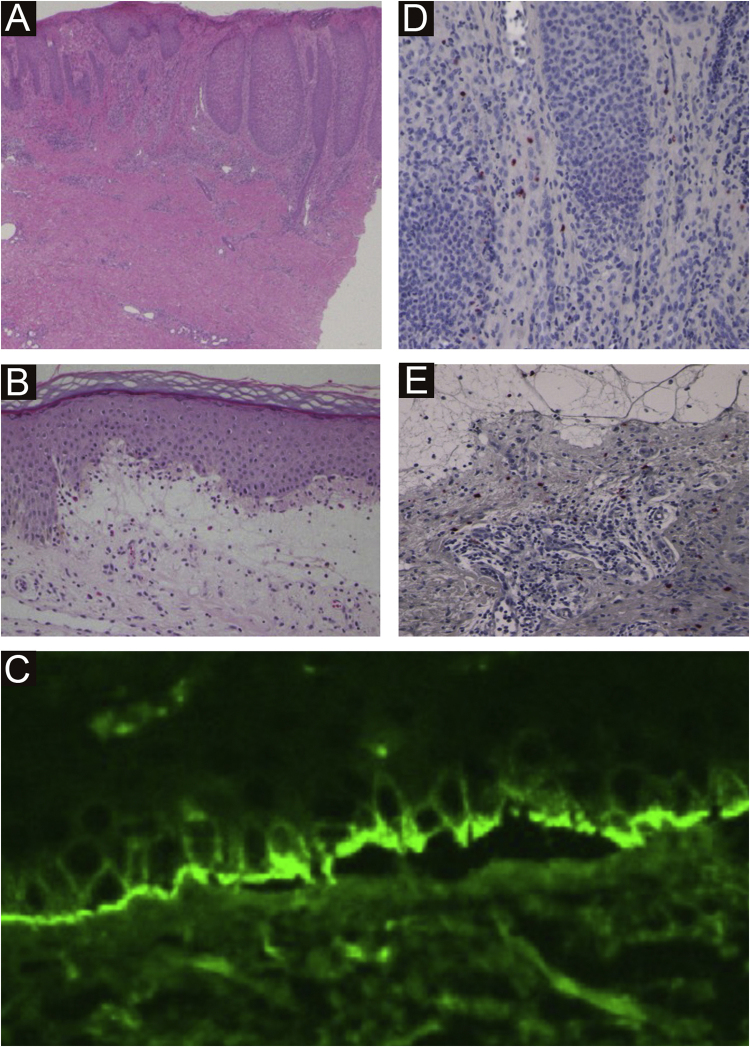
(A) Histological features from nodular prurigo showed irregular acanthosis of the epidermis, inflammatory cell infiltration in the upper dermis, and dermal fibrosis (×40). (B) Histological features from bullous lesion showed subepidermal bulla with eosinophil infiltration (×100). (C) and direct immunofluorescence showed linear deposition of IgG at the basement membrane zon e. BB.1 staining positive basophils in the lesional skin of prurigo nodularis (×200) (D) and BP (×200) (E)

**Figure 3:**
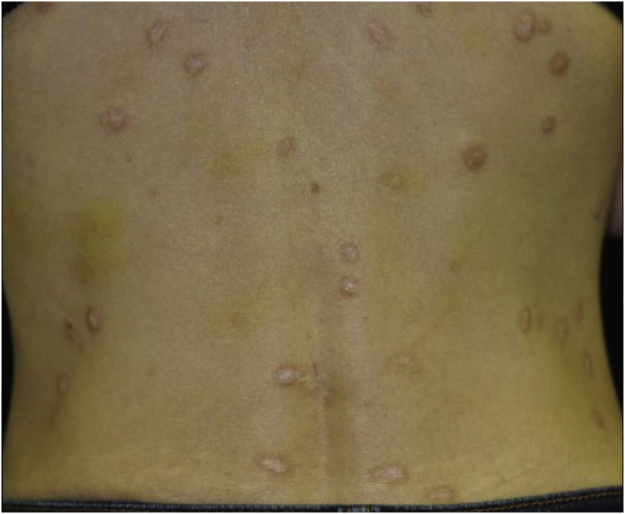
Nodular lesions are dramatically improved after 2 years.

**Figure 4:**
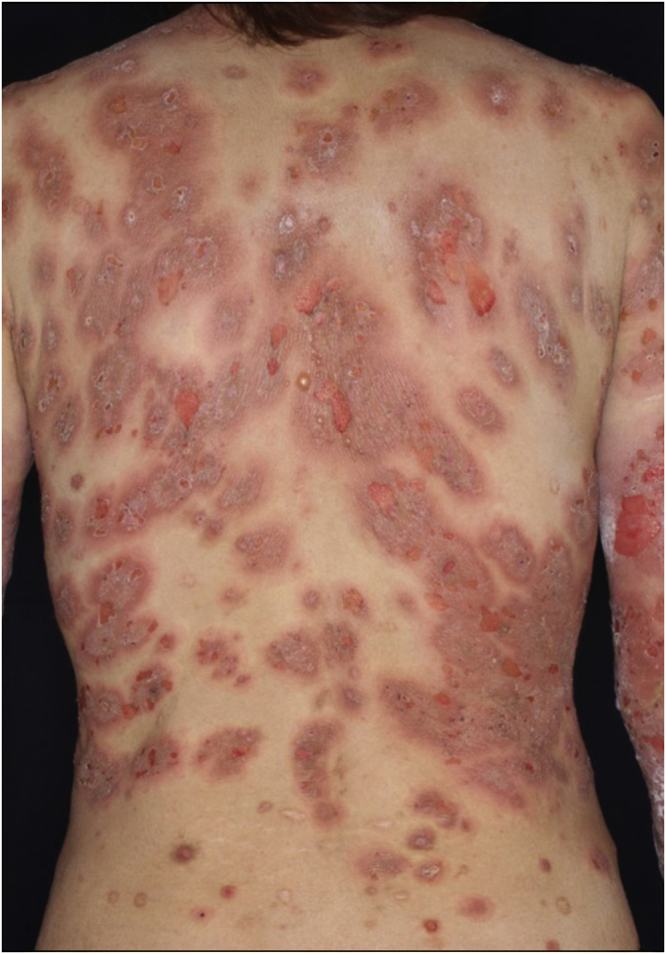
Bullous lesions and erythematous plaques on the trunk

We reported herein a case of BP occurring after the complete resolution of severe PN. A similar case was reported by Yoshimoto et al.,^1^ and their case developed BP 10 months after improvement of PN. They speculated that scratching and local inflammation in PN led to the exposure of neo-epitopes at the basement membrane, resulting in the production of pemphigoid antibodies.^2^ Chen et al.^2^ suggested that the Basement Membrane Zone (BMZ) injuries may have exposed the “hidden” antigens to the immune system, and induced an autoimmune response against the BMZ components. This “epitope spreading” phenomenon may have occurred in our case during the long course.

Recent studies have shown that basophilic infiltration was observed in PN and BP in the activated states.^3^, ^4^ Basophils are one of the major sources of producing Th2-type cytokines leading to increased IgE levels.

In conclusion, basophil was increased in number in both BP and PN lesions and may play an important role in the induction of BP and PN in the present case.

## Financial support

None declared.

## Authors’ contributions

Tomoko Hiraiwa: The study concept and design; data collection, or analysis and interpretation of data; statistical analysis; writing of the manuscript or critical review of important intellectual content; data collection, analysis and interpretation; effective participation in the research guidance; intellectual participation in the propaedeutic and/or therapeutic conduct of the studied cases; critical review of the literature; final approval of the final version of the manuscript.

Natsuko Matsumura: Intellectual participation in the propaedeutic and/or therapeutic conduct of the studied cases; critical review of the literature; final approval of the final version of the manuscript.

Tatsuhiko Mori: Intellectual participation in the propaedeutic and/or therapeutic conduct of the studied cases; critical review of the literature; final approval of the final version of the manuscript.

Nobuyuki Kikuchi: Intellectual participation in the propaedeutic and/or therapeutic conduct of the studied cases; critical review of the literature; final approval of the final version of the manuscript.

Toshiyuki Yamamoto: The study concept and design; statistical analysis; effective participation in the research guidance; critical review of the literature; final approval of the final version of the manuscript.

## Conflicts of interest

None declared.
